# Young Children’s Eating in the Absence of Hunger: Links With Child Inhibitory Control, Child BMI, and Maternal Controlling Feeding Practices

**DOI:** 10.3389/fpsyg.2021.653408

**Published:** 2021-11-16

**Authors:** Kaat Philippe, Claire Chabanet, Sylvie Issanchou, Sandrine Monnery-Patris

**Affiliations:** Centre des Sciences du Goût et de l'Alimentation, AgroSup Dijon, CNRS, INRAE, Université Bourgogne Franche-Comté, Dijon, France

**Keywords:** parental feeding practices, preschoolers, self-regulation of food intake, executive functioning, restriction, food rewards, structural equation modeling

## Abstract

This study aimed to gain a better understanding of the associations between young children’s eating in the absence of hunger (EAH), inhibitory control, body mass index (BMI) and several maternal controlling feeding practices (food as reward, restriction for health, restriction for weight control). In addition, to more properly assess the relationship between children’s and maternal variables, the link between EAH and restriction was explored separately in two directionalities: “child to parent” or “parent to child.” To do this, mothers of 621 children aged 2.00–6.97years (51% boys, *M*=4.11years, *SD*=1.34) filled in a questionnaire with items from validated questionnaires. Structural equation modeling (SEM) was used to analyze the data. The results showed, whatever the directionality considered, a positive association between children’s eating in the absence of hunger and their BMI z-scores. Restriction for health and restriction for weight control were differently linked to EAH and to children’s BMI z-scores. Namely, low child inhibitory control, food as reward and restriction for health were identified as risk factors for EAH. Restriction for weight control was not linked to EAH, but was predicted by child BMI z-scores. Interventions aiming to improve children’s abilities to self-regulate food intake could consider training children’s general self-regulation, their self-regulation of intake, and/or promoting adaptive parental feeding practices.

## Introduction

The prevalence of overweight and obesity in children and adolescents has increased in a large number of countries since the 1980s (GBD 2015 Obesity Collaborators, 2017). World Health Organization (2018) reported that on average almost one in eight children aged seven to eight has obesity in Europe. This is a reason for concern given that childhood obesity has been associated with social, psychological, emotional and health effects both in the short and long terms (for reviews see Reilly et al., 2003; Pulgarón, 2013; Kelsey et al., 2014; Rankin et al., 2016). Stimulating healthy eating habits from an early age could be an important way to prevent overweight and obesity in children, especially as it is known that eating habits established during childhood can persist into adolescence and adulthood (Nicklaus and Remy, 2013).

Young children are believed to have an innate capacity to self-regulate their food intake, by following their internal signals of hunger and fullness (e.g., Birch and Deysher, 1986). As they grow older, environmental factors, such as inappropriate portion sizes, the availability of energy-dense foods and parental controlling food practices could divert children from their internal signals and could cause them to overeat, resulting in an increased risk of weight gain (Birch et al., 2003; Fisher and Kral, 2008; Kral et al., 2012; Frankel et al., 2014; Monnery-Patris et al., 2019). Many studies have examined how the use of controlling feeding practices, in particular restriction and pressure to eat but also food as reward, influences child eating behaviors (e.g., Johnson and Birch, 1994; Fisher and Birch, 2002; Remy et al., 2015; Powell et al., 2017). Overall, the results of these studies indicated a counterproductive effect of these practices as they were linked to or resulted in less adaptive child eating behaviors.

Not only environmental factors, but also children’s temperamental traits play a role in their ability to self-regulate food intake and their weight status. Inhibitory control is an executive functioning process that has been studied extensively in relation to eating behaviors. Inhibitory control refers to the ability to inhibit a dominant behavior or to engage in behavior required for an activity (Posner and Rothbart, 2000). A wide variety of methods exist to measure children’s inhibitory control: both behavioral tasks (e.g., general or food-specific Go/No-Go task, Stroop test, Stop signal task, Peg tapping task) and scales such as the Children’s Behavior Questionnaire (Rothbart et al., 2001) and its variants. In previous studies with children and adolescents, a lower inhibitory control has been linked with binge eating behaviors (Ames et al., 2014; Kittel et al., 2017), higher increases in food enjoyment and food responsiveness (Groppe and Elsner, 2015), lower abilities to self-regulate intake (Tan and Holub, 2011), and a higher body mass index (BMI) or more weight problems (e.g., Nederkoorn et al., 2006, 2012; Graziano et al., 2010; Houben et al., 2014).

An eating behavior reflecting self-regulation of intake that is of interest in relation to children’s weight status is “eating in the absence of hunger” (EAH). EAH refers to children’s susceptibility to eating when satiated if presented with palatable energy-dense foods (Cutting et al., 1999; Fisher and Birch, 2002), and has been associated with increased energy intake (Fisher and Birch, 1999; Birch and Fisher, 2000) and weight status (Fisher and Birch, 2002; Kral et al., 2012; Monnery-Patris et al., 2019). EAH has originally been measured in laboratory settings where children have *ad libitum* free access to foods after a meal and after having reported they were full. EAH referred to the energy intake (number of calories) consumed during the free-access session (Fisher and Birch, 1999). This paradigm is, however, costly and time-consuming, and the ecological validity of the paradigm has been questioned (Madowitz et al., 2014). As a response to these challenges, several questionnaires have been developed to measure EAH in a less costly and more efficient way, and to facilitate longitudinal studies. For example, the Eating in the Absence of Hunger Questionnaire for Children and Adolescence (EAH-C; Tanofsky-Kraff et al., 2008), a self-report for youth aged 6–19years, and a parallel version for parents (EAH-P; Shomaker et al., 2013) have been proposed for English-speaking populations. A French questionnaire for parents has been developed to measure the degree of EAH in children aged 1–5years (Monnery-Patris et al., 2019). Another concept that is of interest in relation to children’s weight status is their appetite (Carnell and Wardle, 2008; Godefroy et al., 2016). Appetite is usually defined as a desire for food, and children with a low appetite usually have a lower weight than children with a high appetite (e.g., Lee and Song, 2007).

Some studies have already investigated possible links between EAH, and the previously mentioned environmental (parental controlling feeding practices) and temperamental factors (inhibitory control). For instance, Rollins et al. (2014) observed that the link between parental controlling feeding practices and EAH was moderated by girls’ level of inhibitory control: more parental restriction for snacks was associated with higher increases in EAH from age 5 to 7 years, but only in girls with a lower inhibitory control. In a longitudinal study with assessments at age 5, 7, 9, 11, 13, and 15years, Anzman and Birch (2009) identified parental restriction as a moderator between girls’ inhibitory control and their BMI: here, a lower inhibitory control was associated with a higher BMI, and this relation was stronger in the presence of higher parental restriction. However, inconsistent results have been reported in the literature for the links between EAH, weight status and controlling feeding practices, and many questions remain. On the one hand, this might be due to the use of different measures for these constructs, as discussed above for EAH and inhibitory control. Different measures have also been used for studying parental controlling feeding practices. To illustrate, in the Child Feeding Questionnaire (Birch et al., 2001), the dimension “restriction” combines the constructs restriction and food as reward, while the Comprehensive Feeding Practices Questionnaire (Musher-Eizenman and Holub, 2007) contains separate dimensions to refer to food as reward and restriction, and even distinguishes between parental motivations/concerns behind the use of restrictive practices; resulting in the dimensions “food as reward,” “restriction for health” and “restriction for weight control.” On the other hand, inconsistent results might be found due to differences in authors’ hypotheses and the associated statistical models and analyses. In fact, in some studies, parental controlling feeding practices were hypothesized to be the explaining variable, while in other studies they were the explained variable or a moderating variable. Small sample sizes in certain studies could also be problematic (Francis and Riggs, 2018).

**Figure 1:**
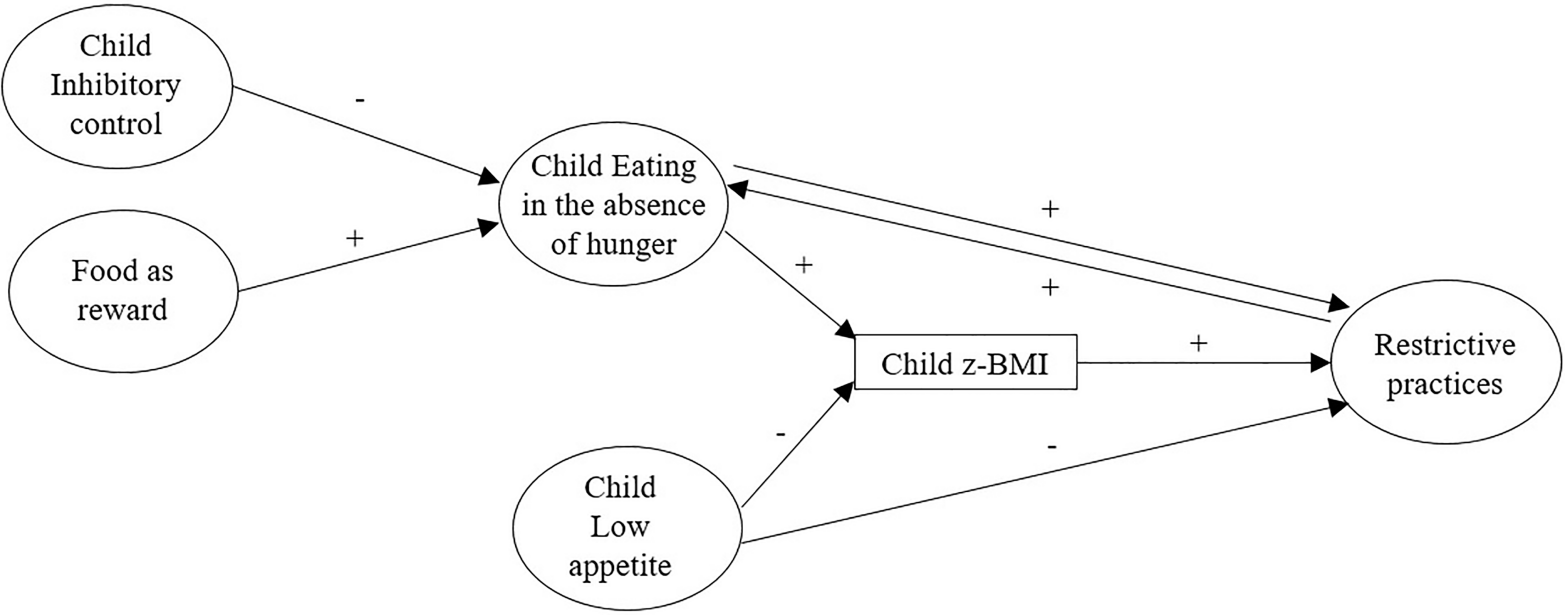
Conceptual model of the study representing the hypotheses. A plus (+) indicates an expected positive relation between constructs, a minus (−) indicates an expected negative relation. The double arrow between eating in the absence of hunger (EAH) and restrictive practices represents two hypotheses that will be tested separately.

Due to its assumed relation with children’s weight status, it is crucial to gain a better understanding of factors that are linked to children’s EAH. Therefore, this study aimed to assess the relationship between EAH and children’s weight status, and to assess variables that could influence EAH in children (see Figure 1). More precisely, this study wanted to assess the influence of variables related to children’s eating behavior, EAH and appetite, on children’s BMI z-score, and the influence of child inhibitory control and maternal controlling feeding practices (food as reward, restriction for weight, and restriction for health) on EAH. In previous literature, maternal restriction has been considered as a cause (Birch et al., 2003) or a consequence (Tan and Holub, 2011) of children’s EAH/self-regulation of eating. Therefore, to take into account these possibilities, both directionalities were considered in this study: an effect of “parent to child,” or of “child to parent.”

It was hypothesized, based on previous studies, that higher levels of EAH and appetite would be linked to higher BMI z-scores in children (e.g., Carnell and Wardle, 2008; Monnery-Patris et al., 2019), and that a lower inhibitory control in children (Nederkoorn et al., 2006, 2012), a higher use of food as reward (Remy et al., 2015), of restriction for health and of restriction for weight control in mothers (Birch et al., 2003; Tan and Holub, 2011) would be linked to higher levels of EAH in children.

## Materials and Methods

### Participants’ Recruitment and Procedure

The recruitment of participants took place as part of a project whose overall aim was to study parental feeding practices and their links with child eating behaviors in France, and which encompassed several research objectives (see, e.g., Philippe et al., 2021). Caregivers were recruited *via* daycare centers and preschools in France, with the use of social media (Facebook, Twitter) and through an internal database (ChemoSens Platform’s PanelSens, CNIL no.1148039). They were invited to complete a hard copy version of the questionnaire or the online version, available on the platform SurveyMonkey. For the study presented in this article, all caregivers fulfilling a mother role for a child aged 2–6years were eligible to participate. They were informed that their participation was voluntary and without compensation. An ethical approval (n°19–591) was granted for the large project by the Institutional Review Board (IRB00003888, IORG0003254, and FWA00005831) of the French Institute of Medical Research and Health, and a study registration was done by the data protection service involved (CNRS).

### Measures

Questionnaires were used to collect data because of several reasons. First of all, they can be used easily in large-scale studies: to recruit a high number of people that are living in different areas. Moreover, a questionnaire may be more relevant than a laboratory setting, since it allows to take into account not only the eating behavior and adjustment of intake during one meal (i.e., short-term compensation), as in experimental settings, but also the pattern over a time period that is longer than just one meal. The same is true for children’s temperament/behavior and parental feeding practices. For this study, questionnaires were selected that were already validated in French for parents of young children.

#### Child Eating Behaviors

##### Low Appetite

The child’s low appetite was measured with three items of the Children’s Eating Difficulties Questionnaire (CEDQ; Rigal et al., 2012). Mothers had to rate their agreement with each of the items [e.g., *My child eats small quantities (even if the food is liked)*] on a five-point Likert scale ranging from (1) “Strongly disagree” to (5) “Strongly agree.” All items are presented in Table 1. A score was calculated for each child by averaging the scores on the three items; a higher score indicated a lower appetite.

**Table 1:** Cronbach’s alphas for dimensions and final item loadings in confirmatory factor analyses (CFA).

Items and related dimensions	Loading
Dimensions concerning the children	
**Appetite^a^ (Cronbach’s alpha=0.85)**	
app1. My child eats small quantities (even if the food is liked).	0.77
app2. My child is a small eater (whatever is served, bad or good).	0.86
app3. My child has a big appetite. (Reversed item)	0.95
**Eating in the absence of hunger^e^ (Cronbach’s alpha=0.66)**	
eah1. If my child is no longer hungry and I offer him something s/he particularly likes, s/he eats it.^b^	0.65
eah2. If my child is no longer hungry and I offer him something s/he particularly like, s/he takes them in order to have them later.^b^ (Reversed item)	Removed
eah3. After s/he has finished his meal, if candies are available and I let him/her, s/he eats it.^b^	0.71
eah4. After s/he has finished his meal, if candies are available and I let him/her, s/he takes them in order to have them later.^b^ (Reversed item)	Removed
eah5. If my child is no longer hungry and I offer him something s/he particularly likes… (*Tick your answer*)^c^	0.69
eah6. After s/he has finished his meal, if candies are available and I let him/her… (*Tick your answer*).^d^	0.73
**Inhibitory control^f^ (Cronbach’s alpha=0.66)**	
ic1. My child can easily stop an activity when s/he is told “no.”	0.64
ic2. My child can wait before entering into new activities if s/he is asked to.	0.82
ic3. My child has trouble sitting still when s/he is told to (at movies, etc.). (Reversed item)	0.42
ic4. My child is capable to follow instructions.	0.61
ic5. My child approaches places s/he has been told are dangerous slowly and cautiously.	0.49
**Dimensions concerning the mothers**	
**Food as reward^b^ (Cronbach’s alpha=0.76)**	
fr1. I offer my child his/her favorite foods in exchange for good behavior.	0.84
fr2. I withhold sweets/dessert from my child in response to bad behavior.	0.72
fr3. I offer sweets (candy, ice cream, cake, pastries) to my child as a reward for good behavior.	0.85
**Restriction for weight control^a^ (Cronbach’s alpha=0.75)**	
restr.w1. I encourage my child to eat less so he/she will not get fat.	0.76
restr.w2. I give my child small helpings at meals to control his/her weight.	0.85
restr.w3. If my child eats more than usual at one meal, I try to restrict his/her eating at the next meal.	0.71
restr.w4. I restrict the food my child eats that might make him/her fat.	Removed
restr.w5. I have to be sure that my child does not eat too many high-fat foods.	Removed
restr.w6. There are certain foods my child should not eat because they will make him/her fat.	0.72
restr.w7. I do not allow my child to eat between meals because I do not want him/her to get fat.	Removed
restr.w8. I often put my child on a diet to control his/her weight.	0.61
**Restriction for health^a^ (Cronbach’s alpha=0.71)**	
restr.h1. I have to be sure that my child does not eat too many sweets (candy, ice cream, cake, or pastries).	Removed
restr.h2. If I did not guide or regulate my child’s eating, s/he would eat too much of his/her favorite foods.	0.72
restr.h3. I have to be sure that my child does not eat too much of his/her favorite foods.	0.63
restr.h4. If I did not guide or regulate my child’s eating, he/she would eat too many snacking foods type cookies, bars chips, sugary foods.	0.80

a *Answer modalities: five-point scale ranging from (1) “Strongly disagree” to (5) “Strongly agree*.”

b *Answer modalities: five-point scale ranging from (1) “Never” to (5) “Always*.”

c *Answer modalities: (1) S/he does not want it, (2) S/he eats a few bites, just to taste it, (3) S/he eats it. Scores have been recoded to (1), (3), (5) to match the scores of items eah1-eah4 (five-point scale)*.

d *Answer modality: (1) S/he does not take any, (2) S/he takes one or two just to taste them, (3) S/he takes a lot. Scores have been recoded to (1), (3), (5) to match the scores of items eah1-eah4 (five-point scale)*.

e *Some original items of this dimension and their answer modalities (Monnery-Patris et al., 2019) were modified for this study, aiming to enable more precise answers. The two original items were: eah1: “If my child is no longer hungry and I offer him something s/he particularly likes… (Tick your answer)” with the answer options (1) S/he does not want it, (2) S/he asks if s/he can have it later, (3) S/he eats a few bites, just to taste it, (4) S/he eats it up.; eah2: “After s/he has finished his meal, if candies are available and I let him/her… (Tick your answer)” with the answer options (1) S/he does not take any, (2) S/he takes them in order to have them later, (3) S/he takes one our two just to taste it, (4) S/he takes a lot*.

f *Answer modalities: seven-point scale ranging from (1) “Very untrue” to (5) “Very true*.”

##### Eating in the Absence of Hunger

The child’s eating in the absence of hunger (EAH) was measured with six items of a recent French questionnaire (Monnery-Patris et al., 2019). Some original items of this dimension and their answer modalities were slightly modified for this study, aiming to enable more precise answers (all items and additional information are presented in Table 1). For four items in this study, mothers had to rate their answer on a five-point scale ranging from (1) “Never” to (5) “Always” (e.g., *If my child is no longer hungry and I offer him something s/he particularly likes, s/he eats it*.). For the two other items, mothers had to identify one of the three answer options that best suited their child’s behavior: e.g., for the item: “After s/he has finished his meal, if candies are available and I let him/her,” they could choose between the options (1) “s/he does not take any,” (2) “s/he takes one or two just to taste them,” or (3) “s/he takes a lot.” The answers to these two last items were recoded to (1), (3), (5) to match the answers of the other items (five-point scale). A score was calculated for each child by averaging the scores on all items; a higher score indicated a higher level of EAH and thus a poorer self-regulation.

#### Child Inhibitory Control

The child’s inhibitory control was measured with five items of the Children’s Behavior Questionnaire Short Form (CBQ; original English version: Putnam and Rothbart, 2006; French-Canadian version: Lemelin et al., 2020). Originally, this Short Form contains six items to measure inhibitory control (e.g., *My child can wait before entering into new activities if s/he is asked to*.). Based on feedback from parents who pretested the questionnaire used for the current study, it was decided to delete one item (i.e., *My child prepares for trips and outings by planning things s/he will need*.). Parents declared that this item was not fully adapted to age range of the children in the current study, as the CBQ was developed for children aged 3–8years while we included children aged 2–6years in the study. Mothers were asked to rate their agreement with each item on a seven-point scale ranging from (1) “Very untrue” to (7) “Very true,” according to their child’s behavior. All items are presented in Table 1. A score was calculated for each child by averaging the scores on all items; a higher score indicated a higher level of inhibitory control.

#### Maternal Controlling Feeding Practices

Maternal use of controlling feeding practices was measured with the Comprehensive Feeding Practices Questionnaire (Musher-Eizenman and Holub, 2007). For this study, the practices of interest were restriction for health (four items, e.g., *If I did not guide or regulate my child’s eating, he/she would eat too many junk foods*), restriction for weight control (eight items, e.g., *I often put my child on a diet to control his/her weight*), and food as reward (three items, e.g., *I offer my child his/her favorite foods in exchange for good behavior*). All items are presented in Table 1. Mothers had to rate their agreement with each item on a five-point scale ranging from (1) “Strongly disagree” to (5) “Strongly agree,” or from (1) “Never” to (5) “Always.” The psychometric properties of this questionnaire have been demonstrated in French samples (Musher-Eizenman and Holub, 2007; Musher-Eizenman et al., 2009). A score was calculated for each parent for each of the three feeding practices by averaging the scores on the corresponding items; a higher score indicated a higher use of the corresponding controlling practice.

#### Anthropometric Data

Mothers were instructed to report the most recent measurements from the child’s medical health book which were carried out by health professionals. If no recent measurements were available, or if the measurements of height and weight were not carried out within a short time span, mothers were instructed to measure and/or weigh the child in light clothes. Children’s BMI (kg/m^2^) was calculated and normed BMI z-scores were calculated using French growth standards for children (Rolland-Cachera et al., 1991, 2002). The child’s birth date was used for a precise calculation of the child’s age.

### Statistical Analyses

R version 3.6.1 (R Core Team, 2019) was used to clean and analyze the data. The significance level was set at *p*<0.05 for all analyses.

#### Data Cleaning and Preliminary Analyses

Questionnaires of mothers were excluded if the child was not aged 2–6.99years, if the child was born premature (<37weeks of gestation), if the child had an illness susceptible of affecting his/her eating behavior (e.g., swallowing problems, food allergies) or if information about one of these aspects was missing. Questionnaires were also excluded if the child’s sex was not provided, if a mother already completed a questionnaire for a sibling, or if there was a high number of missing items. This resulted in the exclusion of 389 questionnaires. A total of 621 questionnaires were maintained for the analyses of the present study: 190 hard copies and 431 online copies.

Confirmatory factor analyses (CFA) with a structural equation modeling (SEM) approach (Bollen, 1989; Kaur et al., 2006) were performed to verify the internal consistency of the scales. First, before conducting the CFA’s, imputation by predictive mean matching was used to account for missing data of the items of interest (the proportion of missing data was lower than 1% for each item). Then, different CFA measurement models were fitted: one for the child eating dimensions, one for child inhibitory control, and one for the maternal feeding practices. According to the fit indices and the estimated loadings, a few items had to be removed: two items for the dimension EAH, one item for restriction for health and two items for restriction for weight control. Finally, Cronbach’s alphas were calculated with the retained items to report the internal consistency of the dimensions; they ranged between 0.66 (EAH; inhibitory control) and 0.85 (appetite). All Cronbach’s alphas, final item loadings in the CFAs and removed items are presented in Table 1.

#### Main Analyses

Scores were calculated for child behaviors and for maternal feeding practices by averaging the scores on the corresponding items. Correlations were calculated to explore the links between the dimensions related to maternal feeding practices (food as reward, restriction for health, and restriction for weight control), child’s inhibitory control, child’s EAH, and child’s BMI z-scores. Simple regressions were also performed to study possible effects of child age and sex on children’s behaviors and maternal practices.

Thereafter, SEM analyses were conducted to assess the structure between these different dimensions, based on our hypotheses derived from past literature. SEM methodology was chosen because it enables to formulate several hypotheses in a global model and to test if the data are in line with the hypotheses. Following the idea that children’s eating behavior influences their BMI z-scores, we hypothesized that EAH (the focus in this study) and appetite would be direct predictors of child BMI z-scores. Then, we assumed that maternal feeding practices (e.g., Birch et al., 2003) and child inhibitory control (Nederkoorn et al., 2006, 2012) could influence children’s EAH, but not their appetite since this is considered as a fairly stable eating trait in children (Farrow and Blissett, 2012). In addition, previous studies have pointed out that children’s EAH and (maternal perceptions of) their weight status and appetite could also predict maternal restrictive practices (Webber et al., 2010; Tan and Holub, 2011). We thus considered that restriction could be either a cause or a consequence of EAH. Finally, since we expected a stronger link with child BMI z-scores for restriction for weight control than for restriction for health, these two forms of restriction were considered in separated models.

Thus, we ran separate models for restriction for weight control and restriction for health, and two types of models were estimated to take into account the possible different directionalities between EAH and maternal restriction (effects of “child to parent” and of “parent to child”). This resulted in four separate models: (1A) “child to parent” with restriction for weight control, (1B) “parent to child” with restriction for weight control, (2A) “child to parent” with restriction for health, and (2B) “parent to child” with restriction for health.

All SEM analyses were conducted using the R package lavaan 0.6–7 (Rosseel, 2012). All items except child BMI z-score were declared as ordered. For all models, only data of participants without missing child BMI z-score were used. The root mean square error of approximation (RMSEA), the comparative fit index (CFI) and the Tucker–Lewis Index (TLI) were used to evaluate the fit of each model. A low RMSEA and high CFI and TLI indicate a good fit (cut-offs: acceptable fit: 0.08 for RMSEA, 0.95 for CFI and TLI; good fit: 0.05 for RMSEA, 0.97 for CFI and TLI; Schermelleh-Engel et al., 2003). As models 1B and 2B present cyclic structures (with a loop between EAH – z-BMI – restriction – EAH), the R package SEMID_0.3.2 was used to verify if these structures were identifiable. The codes used in R for the SEM analyses can be consulted on Zenodo,^1^ together with the data set generated for this study, and the French items used. A metadata file provides information about the published data set and accompanying documents.

## Results

### Participants’ Characteristics

Mothers of 621 children aged 2.00–6.97years (51% boys, mean age=4.11years, *SD*=1.34) participated in this study. The characteristics of the mothers can be found in Table 2. According to maternal reports of child weight and height, 11% of children in our sample were underweight (z-BMI<−2), 71% had a normal weight (−2≤z-BMI<1), 10% were at risk for overweight (1≤z-BMI<2), 5% had overweight (2≤z-BMI<3), and 2% had obesity (z-BMI>3; weight categories according to World Health Organization, 2006). Most children (87%) lived with both parents, 5% of children were in a co-parenting situation, and 8% of children lived with their mother only or with their mother and her partner.

**Table 2:** Mothers’ characteristics.

Characteristics	Mothers (*N*=621)		*N*	%
Hard copy/Online participation	190/431	31/69
Age, mean (SD)	35.26 (4.50)	
**Weight status^a^:**		
Underweight (BMI<18.5kg/m^2^)	27	4
Normal weight (18.5≤BMI<25kg/m^2^)	368	61
Overweight (25≤BMI<30kg/m^2^)	132	22
Obesity (BMI≥30kg/m^2^)	77	13
**Level of education:**		
No diploma	8	1
A level or a high-school diploma/degree	44	7
Diploma of higher education or 12th grade	77	13
Three-year university degree	122	20
Master’s degree or Master 2	225	37
Higher than a Master 2 (PhD, medical studies)	135	22
**Work status:**		
Working (part-time or full-time)	477	78
Unemployed, job seeker	41	7
Student	9	1
Other (e.g., parental leave, parent at home)	50	14
**Perception of financial situation:**		
You cannot make ends meet without going into debt	6	1
You get by but only just	37	6
Should be careful	152	25
It’s OK	276	46
At ease	135	22

a *Mothers’ height and weight, needed for BMI calculations (kg/m^2^), were self-reported*.

### Descriptive Statistics

Mean scores of the study variables, SDs, as well as Spearman correlation coefficients between each other are presented in Table 3. Significant positive correlations were observed between the three maternal controlling feeding practices (food as reward, restriction for health, restriction for weight control). EAH of the child was positively linked to food as reward, restriction for health, child BMI z-score, and negatively linked to child inhibitory control. No significant link was observed between EAH and restriction for weight control. Both types of restrictions and child low appetite were significantly linked to the child’s BMI z-score.

**Table 3:** Spearman correlations, means, and SDs for study variables.

		Variables							Mean (SD)
		1	2	3	4	5	6	7	
**Maternal feeding practices:**									
Food as reward^a^	1	-							1.68 (0.75)
Restriction for health^a^	2	0.22^***^	-						3.08 (1.00)
Restriction for weight control^a^	3	0.18^***^	0.37^***^	-					1.66 (0.64)
**Child behaviors and BMI:**									
Low appetite^a^	4	0.05	0.02	−0.05	-				2.52 (1.08)
Eating in the absence of hunger^a^	5	0.18^***^	0.38^***^	0.04	−0.07	-			3.10 (0.86)
Child inhibitory control^b^	6	−0.09^*^	−0.16^***^	−0.07	0.04	−0.15^***^	-		5.06 (1.01)
Child BMI z-score	7	0.08	0.10^*^	0.17^***^	−0.19^***^	0.09^*^	−0.07	-	−0.22 (1.49)

a *Answer scale ranges from 1 to 5*.

b *Answer scale ranges from 1 to 7*.

* *Significance level: p<0.05*;

*** *Significance level: p<0.001*.

In addition, the mean scores indicated that restriction for health is a commonly used feeding practice among French mothers of children aged 2–6years. Food as reward and restriction for weight control are used to a lesser extent.

Furthermore, simple regression analyses indicated that child sex and child age were significant predictors for a number of child behaviors and maternal feeding practices. Girls showed higher levels of inhibitory control than boys (*β*=+0.31; *t*=3.86; *p*<0.001), and a lower appetite (*β*=+0.34; *t*=3.94; *p*<0.001). Children’s inhibitory control increased significantly with age (*β*=+0.10; *t*=3.34; *p*<0.001), children showed a lower appetite with age (*β*=+0.11; *t*=3.32; *p*<0.001), and mothers reported using more food as reward (*β*=+0.06; *t*=2.77; *p*=0.006) and restriction for weight control (*β*=+0.05; *t*=2.39; *p*=0.017) with an increasing age of the child.

### Structural Equation Models

Four different structural models were evaluated, of which two models included restriction for weight control (model 1A and 1B) and two models included restriction for health (model 2A and 2B). The A-models included the effect of “child (EAH) to parent (restriction),” while the B-models included the effect of “parent (restriction) to child (EAH).” For these models, the data of 541 participants were used (80 children had a missing BMI z-score).

**Figure 2:**
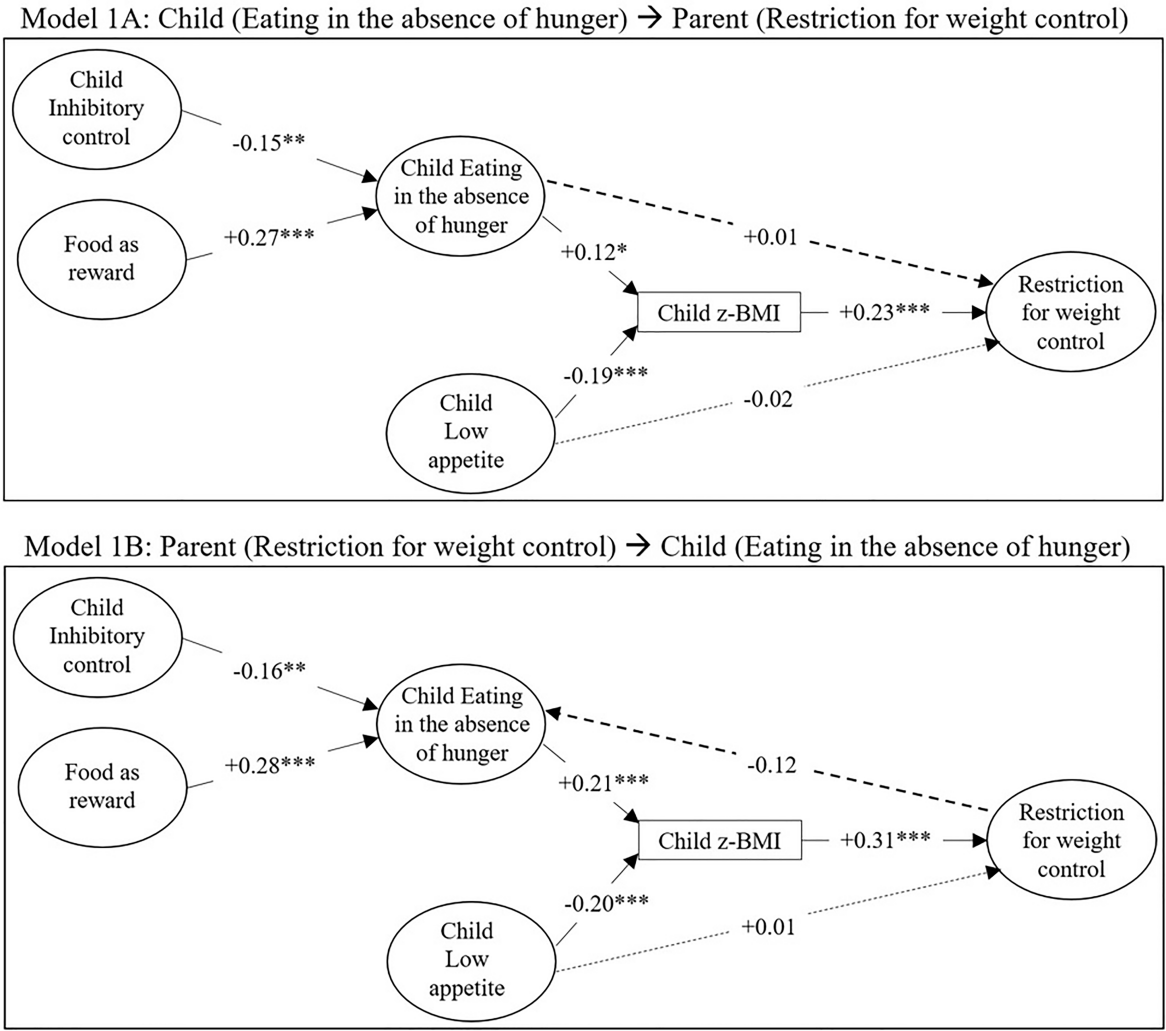
Structural models for the associations between parental feeding practices (restriction for weight control, food as reward), child inhibitory control, child EAH, child low appetite and child body mass index (BMI) z-score. Numbers indicate standardized coefficients, solid lines indicate significant coefficients (^*^p<0.05; ^**^p<0.01; and ^***^p<0.001), and dashed lines indicate non-significant coefficients. The correlations between the exogenous latent variables (food as reward, inhibitory control, low appetite) are not visualized here. Model 1A: model from child’s EAH to mother’s restriction for weight control. Model 1B: model from mother’s restriction for weight control to child’s EAH.

**Figure 3:**
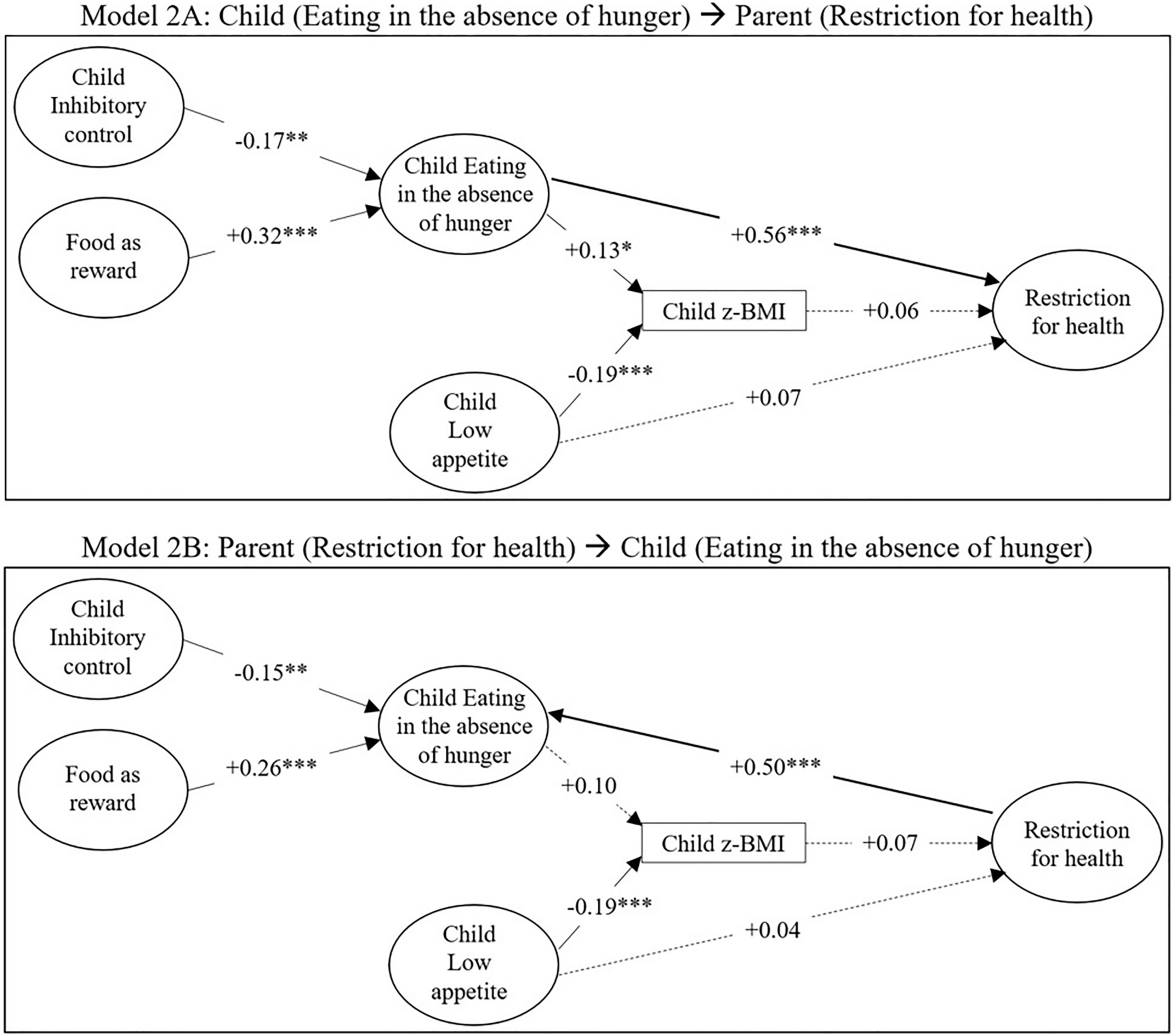
Structural models for the associations between parental feeding practices (restriction for health, food as reward), child inhibitory control, child EAH, child low appetite and child BMI z-score. Numbers indicate standardized coefficients, solid lines indicate significant coefficients (^*^p<0.05; ^**^p<0.01; and ^***^p<0.001), and dashed lines indicate non-significant coefficients. The correlations between the exogenous latent variables (food as reward, inhibitory control, low appetite) are not visualized here. Model 2A: model from child’s EAH to mother’s restriction for health. Model 2B: model from mother’s restriction for health to child’s EAH.

Figures 2, 3 represent the structural part of the models, that is to say the links between the latent variables, respectively, with restriction for weight control and with restriction for health. The corresponding parameters (regressions and covariances) are presented in Tables 4, 5 for models 1A and 1B, and in Tables 6, 7 for models 2A and 2B. All models were identifiable and showed an acceptable fit (see footnote Tables 4–7), so neither of the two directionalities hypothesized could be rejected.

**Table 4:** SEM model 1A: parameter estimates, SE, *z*-values, value of *p*, and standardized estimates (i.e., completely standardized solutions) for regression parameters, and correlations between exogenous latent variables.

Structural regression coefficients	Estimate	SE	*z*-value	*p*	Std. estimate
**Eating in the absence of hunger**					
Child inhibitory control	−0.144	0.050	−2.857	0.004	−0.150
Food as reward	0.211	0.044	4.789	<0.001	0.274
**Child z-BMI**					
Eating in the absence of hunger	0.283	0.113	2.498	0.012	0.120
Low appetite	−0.344	0.079	−4.379	<0.001	−0.189
**Restriction for weight control**					
Child z-BMI	0.126	0.027	4.740	<0.001	0.234
Low appetite	−0.015	0.048	−0.321	0.748	−0.016
Eating in the absence of hunger	0.009	0.074	0.120	0.905	0.007
**Correlations between exogenous latent variables**					
	Food as reward	Child inhibitory control	Low appetite		
Food as reward	-				
Child inhibitory control	−0.113	-			
Low appetite	0.098	0.077	-		

*Robust model fit indexes: RMSEA [90% CI]=0.050 [0.044; 0.056], CFI=0.957, TLI=0.950*.

**Table 5:** SEM model 1B: parameter estimates, SE, *z*-values, value of *p*, and standardized estimates (i.e., completely standardized solutions) for regression parameters, and correlations between exogenous latent variables.

Structural regression coefficients	Estimate	SE	*z*-value	*p*	Std. estimate
**Eating in the absence of hunger**					
Child inhibitory control	−0.149	0.051	−2.929	0.003	−0.155
Food as reward	0.216	0.044	4.892	<0.001	0.281
Restriction for weight control	−0.094	0.050	−1.871	0.061	−0.119
**Child z-BMI**					
Eating in the absence of hunger	0.475	0.131	3.636	<0.001	0.205
Low appetite	−0.354	0.079	−4.506	<0.001	−0.198
**Restriction for weight control**					
Child z-BMI	0.166	0.031	5.312	<0.001	0.305
Low appetite	0.013	0.049	0.275	0.783	0.014
**Correlations between exogenous latent variables**					
	Food as reward	Child inhibitory control	Low appetite		
Food as reward	-				
Child inhibitory control	−0.113	-			
Low appetite	0.101	0.076	-		

*Robust model fit indexes: RMSEA [90% CI]=0.048 [0.042; 0.055], CFI=0.960, TLI=0.953*.

**Table 6:** SEM model 2A: parameter estimates, SE, *z*-values, value of *p*, and standardized estimates (i.e., completely standardized solutions) for regression parameters, and correlations between exogenous latent variables.

Structural regression coefficients	Estimate	SE	*z*-value	*p*	Std. estimate
**Eating in the absence of hunger**					
Child inhibitory control	−0.157	0.047	−3.306	0.001	−0.174
Food as reward	0.235	0.042	5.530	<0.001	0.323
**Child z-BMI**					
Eating in the absence of hunger	0.316	0.122	2.593	0.010	0.126
Low appetite	−0.345	0.078	−4.400	<0.001	−0.190
**Restriction for health**					
Child z-BMI	0.032	0.022	1.472	0.141	0.063
Low appetite	0.062	0.044	1.429	0.153	0.068
Eating in the absence of hunger	0.708	0.082	8.635	<0.001	0.555
**Correlations between exogenous latent variables**					
	Food as reward	Child inhibitory control	Low appetite		
Food as reward	-				
Child inhibitory control	−0.114	-			
Low appetite	0.108	0.073	-		

*Robust model fit indexes: RMSEA [90% CI]=0.044 [0.037; 0.051], CFI=0.972, TLI=0.966*.

**Table 7:** SEM model 2B: parameter estimates, SE, *z*-values, value of *p*, and standardized estimates (i.e., completely standardized solutions) for regression parameters, and correlations between exogenous latent variables.

Structural regression coefficients	Estimate	SE	*z*-value	*p*	Std. estimate
**Eating in the absence of hunger**					
Child inhibitory control	−0.137	0.048	−2.848	0.004	−0.149
Food as reward	0.191	0.042	4.549	<0.001	0.258
Restriction for health	0.391	0.049	8.016	<0.001	0.497
**Child z-BMI**					
Eating in the absence of hunger	0.253	0.137	1.855	0.064	0.103
Low appetite	−0.347	0.078	−4.438	<0.001	−0.191
**Restriction for health**					
Child z-BMI	0.038	0.028	1.372	0.170	0.074
Low appetite	0.039	0.049	0.797	0.425	0.041
**Correlations between exogenous latent variables**					
	Food as reward	Child inhibitory control	Low appetite		
Food as reward	-				
Child inhibitory control	−0.113	-			
Low appetite	0.113	0.072	-		

*Robust model fit indexes: RMSEA [90% CI]=0.059 [0.053; 0.066], CFI=0.949, TLI=0.939*.

In all four models, a negative association was found between child inhibitory control and child EAH, meaning that higher levels of inhibitory control were linked to less EAH. Food as reward was also consistently positively associated with EAH. Furthermore, child low appetite was consistently negatively associated with child BMI z-score, and EAH was positively associated with child BMI z-score, except in model 2B (standardized estimate=0.10; *p*=0.064).

Figure 2 shows that restriction for weight control was only significantly associated with child BMI z-score: a higher BMI z-score was linked to more restriction for weight control. In contrast, Figure 3 shows that restriction for health was unrelated to child BMI z-score. While a strong association was observed between restriction for health and child EAH in both the “child to parent” (2A) and the “parent to child” (2B) model (Figure 3), restriction for weight control was not significantly associated with EAH. Thus, for restriction for weight control, only an indirect link was observed with child EAH *via* child BMI z-score.

## Discussion

Using a large sample of French mothers, this study attempts to unravel the associations between preschoolers’ EAH, inhibitory control, BMI z-score and different maternal controlling feeding practices. The SEM models aiming to estimate these associations were so constructed based on the idea that child weight is a result of children’s eating behavior, and that children’s eating behavior (EAH) is influenced by parental feeding practices and child temperament (Davison and Birch, 2001). In separate models, this study also wanted to take into account the possibility that parental feeding practices are influenced by child eating behavior (Birch et al., 2003; Jansen et al., 2018).

In line with previous studies (Fisher and Birch, 2002; Kral et al., 2012; Monnery-Patris et al., 2019), we observed a significant positive link between children’s EAH and their BMI z-scores. This suggests that as early as the preschool period, poorer abilities to self-regulate food intake could be associated with overeating and could represent a risk of weight gain and for overweight or obesity in the longer run. We also observed that children’s temperament can play a role in their vulnerability toward difficulties with self-regulation of eating. Previous studies have already linked the children’s level of inhibitory control with their eating behavior and self-regulation of intake (e.g., Tan and Holub, 2011), even though the results have sometimes been inconsistent (Francis and Riggs, 2018). Our results seem to confirm that higher levels of inhibitory control could act as a protective factor in relation to eating in the absence of hunger, or vice versa that lower levels of inhibitory control could induce a vulnerability.

The results further indicated that environmental factors, specifically parental feeding practices, were linked to child EAH: both food as reward and restriction for health were significantly positively associated with EAH. One could argue that food as reward is mainly a parent-centered feeding practice; meaning that parents use food rewards in exchange for good behavior of the child, regardless of the child’s eating behavior or eating temperament. For restriction for health, we explored the relation with EAH in two directions (“child to parent” or “parent to child”). In both models, and thus both directions, a significant association was observed. These results could suggest a bidirectional relationship, beyond the scope of the present paper, according to which poor self-regulation in the child might stimulate parents to impose restrictive measures, which in turn, could reinforce the child’s poor self-regulation and divert them from their sensitivity to satiety cues. This bidirectional link was previously already suggested by Bergmeier et al. (2014). Longitudinal studies are, however, needed to further explore these possible bidirectional links between controlling feeding practices and children’s self-regulation of eating. For restriction for weight control, no direct link with EAH was observed in this study, only an indirect link *via* child BMI z-scores. Based on this finding, we think that restriction for weight control could be mainly a child-centered practice: this practice could be dominantly implemented by parents based on the child’s weight status and parental concerns related to this. Accordingly, Musher-Eizenman and Holub (2007) reported that restriction for weight control was significantly linked with parental concerns about the child being overweight (positive link) and concerns about the child being underweight (negative link). The absence of a link between restriction for weight control and EAH is in line with the results of Tan and Holub (2011), but not with those of Musher-Eizenman and Holub (2006), who found that maternal restriction for weight control significantly predicted preschoolers’ EAH. These mixed results could be due to sampling differences, but also due to the use of different measures for children’s self-regulation of eating. For this study and the study of Tan and Holub (2011), parent-reported questionnaires were used, while Musher-Eizenman and Holub (2006) used a behavioral external eating task in a childcare center. This could indicate that both types of measures might tap into different aspects of children’s self-regulation of eating (Tan and Holub, 2011). Moreover, we found that restriction for health was linked to EAH whereas restriction for weight control was not. Even if we cannot give a definite explanation, it is interesting to mention that the items representing restriction for health tap mainly into the types of foods that are restricted (i.e., unhealthy, well-liked foods), while the items representing restriction for weight control (after the removal of certain items based on the fit indices of the CFA’s) tap mainly into the restriction of the amount of the foods (see Table 1). In our study, not only the motivations linked to restriction were thus different, but also the type of restriction. This could indicate that limiting the access to certain types of foods has a stronger link with self-regulation of eating than limiting merely the amount of intake of these foods. Accordingly, previous studies found that prohibiting the intake of certain types of foods leads to an increased desire for and consumption of these foods when granted access to Jansen et al. (2007, 2008).

Overall, our results seem to indicate that factors on both child and parent levels contribute to children’s self-regulation of eating (EAH) and associated weight status, and this already at preschool age. They give rise to the idea that, for children, it could be important to guide them from a very young age in maintaining (or developing) adaptive self-regulation abilities for food intake and to avoid EAH. Parents and schools could play an important role in encouraging children to listen to their inner sensations of hunger and fullness for intake and in modeling these strategies. A limited number of intervention programs exist for children to promote a better self-regulation of eating. They include, for example, appetite awareness trainings, teach concepts of hunger and fullness (e.g., Johnson, 2000; Boutelle et al., 2011; Bloom et al., 2013), or they combine educational materials for parents with an interactive character-based technology platform for the child (Reigh et al., 2020). Some studies also suggest that children could benefit from interventions that train their inhibitory control (e.g., Jiang et al., 2016). However, studies with preschoolers are scarce (e.g., Graziano and Hart, 2016; Lumeng et al., 2017) and with varying results, especially in relation to the food domain (self-regulation of eating) and weight status. More research is needed in this domain. Furthermore, for parents, our results suggest that it is preferable to limit the use of controlling feeding practices, which is in accordance with conclusions in previous studies (Vaughn et al., 2016). In addition to discouraging the use of controlling practices in parents, it could be beneficial to stimulate the use of alternative feeding practices, such as structure-related practices (Rollins et al., 2016; Vaughn et al., 2016). These practices present a certain type of parental control, but in a non-coercive way: they encompass consistent rules and boundaries around eating (e.g., about what, when and where to eat), and are believed to facilitate children’s competences and to promote the adoption of healthy eating behaviors (Jansen et al., 2014; Vaughn et al., 2016). They have also been found beneficial for children’s self-regulation of eating (Frankel et al., 2018). A certain level of parental control in the form of limits, structure and routines could enable children to act autonomously within these predefined boundaries, which might stimulate them to maintain or adopt adaptive strategies to self-regulate their intake.

## Limitations and Strengths

Several limitations should be noted for the current study. First, the cross-sectional design limits the results to mere associations. Longitudinal studies are necessary for studying the causality of the relationships. It is worthy to note, though, that this study did not aim to draw conclusions regarding causality between restriction and EAH, but merely wanted to take into account the possibility of a “child to parent” or a “parent to child” effect. Second, maternal controlling feeding practices were self-reported and might be subject to a social desirability bias. Third, child inhibitory control and EAH were not observed directly but were mother-reported, and might thus be influenced by parental beliefs and perceptions. In two studies, mothers were found to rate the self-regulation of eating of their child higher than fathers did, suggesting the vulnerability to subjectivity of parent-reports of self-regulation (Frankel et al., 2018; Frankel and Kuno, 2019). Parents might have difficulties to report on aspects of self-regulation of eating because these behaviors reflect children’s inner sensations which could be difficult to read. Last, children’s weight and height were mother-reported and the researchers did not know if the measurements were performed by health professionals or not. The quality of the measurements could therefore vary. Taken together, these limitations should be kept in mind when interpreting the results of this study. It would be interesting to conduct a study with data gathered at different time points to properly assess the directionality between the parent and child constructs. In addition, it would be preferable to combine observational and declarative measures to cross-validate the measures. It is also good to take into account the fact that a model is always a simplified representation of the relationships between different variables. For the aim of this study, a number of variables were selected in order to discuss how they relate to each other. Obviously, there are other variables (e.g., maternal weight status, sociodemographic variables) that could be of interest in relation to parental practices and child EAH and BMI. These associations could be explored in future studies.

This study also presents a number of strengths. A first and important strength of this study is its large sample size. Second, this study presents results of a French population which expands the results of studies mainly conducted in the United States. Third, distinct dimensions were used for different parental controlling practices (food as reward, restriction for health, and restriction for weight control) which, sometimes, have been used in combined, overarching dimensions in the past, resulting in mixed results. These distinctions enabled us to obtain a better understanding of the relations between these practices and child behaviors and BMI, and clearly showed that these restrictive practices should be studied as separate dimensions. Last, this study is original in its design by combining temperamental and environmental dimensions that could be linked to child self-regulation and BMI, and by exploring possible different directionalities in separate SEM models.

## Conclusion

In sum, the results of the current study showed a link between young children’s self-regulation of eating and their BMI, identifying EAH as a possible risk factor for the development of weight problems. Both temperamental traits (inhibitory control) and environmental factors (maternal controlling feeding practices) were associated with EAH, and restriction for health and restriction for weight control were linked differently to EAH and to children’s BMI z-scores. Beyond the scope of this study, we think that interventions could focus on improving children’s abilities to self-regulate intake, promoting inhibitory control or promoting adaptive parental feeding practices. It could also be of interest to take on a systemic approach in future interventions in which different actions are combined. These interventions could, for example, propose trainings for children to improve their general and food-related self-regulation. In addition, trainings could guide caregivers in adopting responsive behaviors to their children’s appetite and satiation cues, and in using structure-related parental feeding practices.

This study provided additional insight into the relationships between EAH, BMI, inhibitory control and different maternal feeding practices, but it is important to note that this study focused specifically on maternal feeding practices. Future studies with a large number of fathers are needed to replicate or refute the current results with mothers, as Frankel and Kuno (2019) showed that results regarding the relationship between restrictive feeding practices and children’s self-regulation in eating from mother-only samples should not automatically be generalized to all parents.

## Data Availability Statement

The original contributions presented in the study are publicly available. This data can be found here: https://zenodo.org/record/4436613#.X_8IeuhKi71.

## Ethics Statement

The studies involving human participants were reviewed and approved by Institutional Review Board (IRB00003888, IORG0003254, and FWA00005831) of the French Institute of Medical Research and Health. Written informed consent for participation was not required for this study in accordance with the national legislation and the institutional requirements.

## Author Contributions

KP, SI, SM-P, and CC conceptualized the study. KP and CC conducted all analyses. KP is first author, she drafted a first version of the manuscript, SI, SM-P, and CC thereafter contributed to editing the manuscript. All authors contributed to the article and approved the submitted version.

## Funding

This work was supported by the European Union’s horizon 2020 research and innovation program (Marie Sklodowska-Curie grant agreement No 764985: EDULIA project).

## Conflict of Interest

The authors declare that the research was conducted in the absence of any commercial or financial relationships that could be construed as a potential conflict of interest.

## Publisher’s Note

All claims expressed in this article are solely those of the authors and do not necessarily represent those of their affiliated organizations, or those of the publisher, the editors and the reviewers. Any product that may be evaluated in this article, or claim that may be made by its manufacturer, is not guaranteed or endorsed by the publisher.

## Footnotes

^1^ https://zenodo.org/record/4436613#.X_8IeuhKi71
